# A Case of Laparoscopic Transabdominal Pre-peritoneal Hernia Repair Using a 3D Mesh Inversion Technique for Bilateral Obturator and Femoral Hernias After Incarcerated Obturator Hernia Reduction

**DOI:** 10.7759/cureus.74493

**Published:** 2024-11-26

**Authors:** Yoshiyuki Chiba, Kiichi Sugimoto, Naoki Negami, Yasunori Ishido, Hiroyuki Sugo

**Affiliations:** 1 Department of General Surgery, Juntendo University Nerima Hospital, Tokyo, JPN; 2 Department of Coloproctological Surgery, Juntendo University Faculty of Medicine, Tokyo, JPN; 3 Department of General Surgery, Saiseikai Kawaguchi General Hospital, Saitama, JPN

**Keywords:** 3d mesh inversion technique, femoral hernia, general surgery, obturator hernia, tapp

## Abstract

An obturator hernia (OH) is a rare type of hernia that accounts for a very small proportion of all hernias and cases of small bowel obstruction. This condition predominantly affects older, underweight individuals, with the vast majority of patients being women. Laparotomy with simple suture closure of the defect is commonly used as surgical treatment for OH. However, the closeness of the obturator nerve to the obturator defect causes difficulty in observing this nerve due to the deep operative field in laparotomy. Thus, transabdominal pre-peritoneal hernia repair (TAPP) has advantages over an open approach, and TAPP is now commonly performed for OH. In the case described here, an 86-year-old female patient presented with lower abdominal pain and vomiting. Abdominal computed tomography revealed a right-sided OH causing intestinal obstruction, and the patient was referred to our hospital. Her medical history included hypertension, dementia, and an artificial head replacement for a left femoral neck fracture. Laboratory tests showed elevated white blood cell (WBC) of 13,700/μL, but other results were normal. Manual reduction of the hernia was successful, leading to symptom improvement, and the patient was admitted for observation. She was discharged on day three after admission. After one month, laparoscopic TAPP was performed. Bilateral OHs and femoral hernias were observed. Using a recently proposed 3D mesh inversion technique, the mesh was fitted anatomically for the OH. This is the first reported case of elective TAPP using a 3D MAX^TM ^Light Mesh (Bard, Warwick, RI, US) with an inversion technique for bilateral OH and femoral hernia after incarcerated OH reduction.

## Introduction

An obturator hernia (OH) occurs through the obturator canal, which is 2-3 cm long and 1 cm wide [1]. Among hernias, 0.07% to 1% are diagnosed as OH. Inguinal or femoral hernias typically cause pain in the groin area, whereas OHs present with nonspecific symptoms, often mimicking bowel obstruction. OH accounts for 0.2%-1.6% of all cases of mechanical small bowel obstruction [2]. OH tends to be more common in older women, especially those who are thin, and women account for >97% of affected patients [3]. Surgery is the standard of care for OH, and laparotomy with simple suture closure of the defect is commonly used. However, the closeness of the obturator nerve to the obturator defect creates difficulty with observation in the laparotomy operative field. Transabdominal pre-peritoneal hernia repair (TAPP) for OH has several advantages over an open approach and is now commonly performed [4]. Sasa et al. proposed laparoscopic inverted placement of a 3D MAX^TM^ Light Mesh (Bard, Warwick, RI, US) for an OH, and we were able to make the mesh fit anatomically using this inversion technique for an OH. Because the shape of the mesh could be maintained, it was possible to coat the hernia orifice by fixing the mesh at a minimum level [5]. Here, we present the first case of elective TAPP using a 3D mesh with an inversion technique for bilateral OH and femoral hernia after incarcerated OH reduction.

## Case presentation

**Figure 1:**
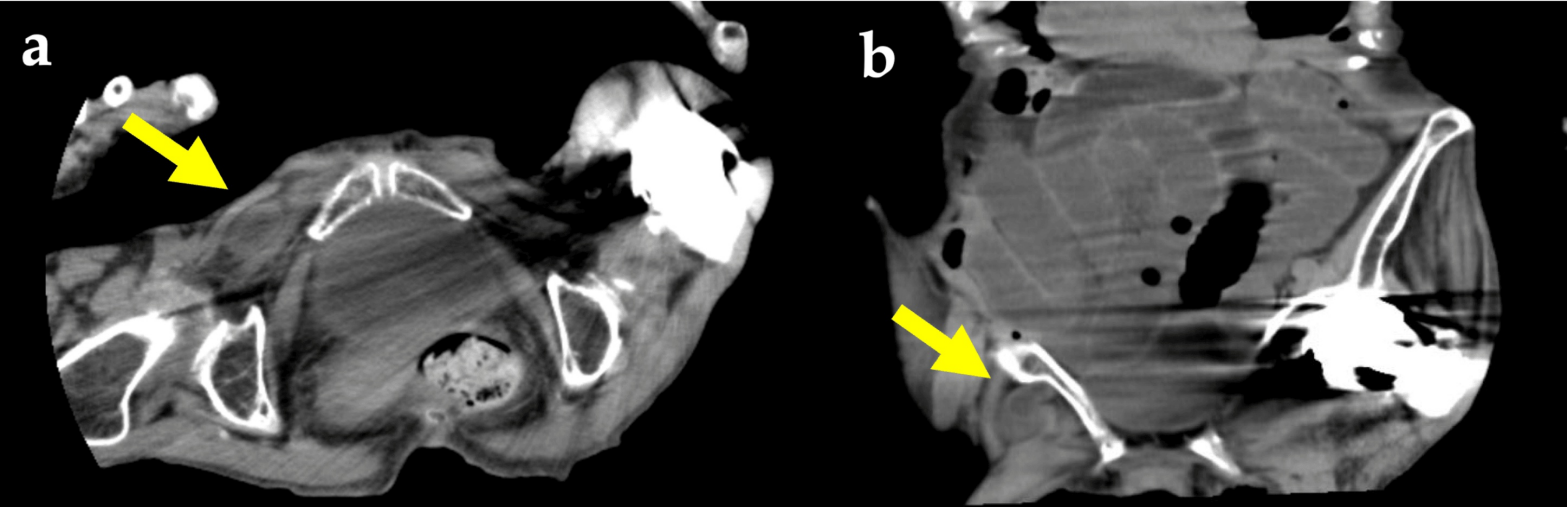
Abdominal computed tomography scan showing right obturator hernia incarceration (arrows): (a) axial view; (b) coronal view.

An 86-year-old female patient presented to her general practitioner complaining of lower abdominal pain and vomiting. Her medical history included hypertension, dementia, and artificial head replacement for a left femoral neck fracture. Clinical symptoms included abdominal distension and a 3-cm mass palpable in the groin area. Laboratory tests showed an elevated white blood cell (WBC) count of 13,700/μL, but C-reactive protein (CRP) (0.04 mg/dL) and blood gas analysis were normal. A computed tomography (CT) scan by a family doctor revealed that the bowel was incarcerated in the right OH and the proximal side of the bowel was dilated, suggesting bowel obstruction (Figure 1).

**Figure 2:**
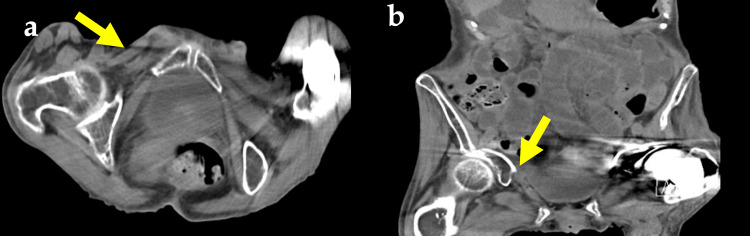
Abdominal computed tomography scan showing obturator hernia reduction (arrows): (a) axial view; (b) coronal view.

On the basis of these findings, we made a diagnosis of incarcerated OH. We attempted manual reduction of the incarcerated OH and were successful. After reduction, lower abdominal pain and vomiting symptoms improved, and CT showed that the OH was no longer incarcerated (Figure 2).

**Figure 3:**
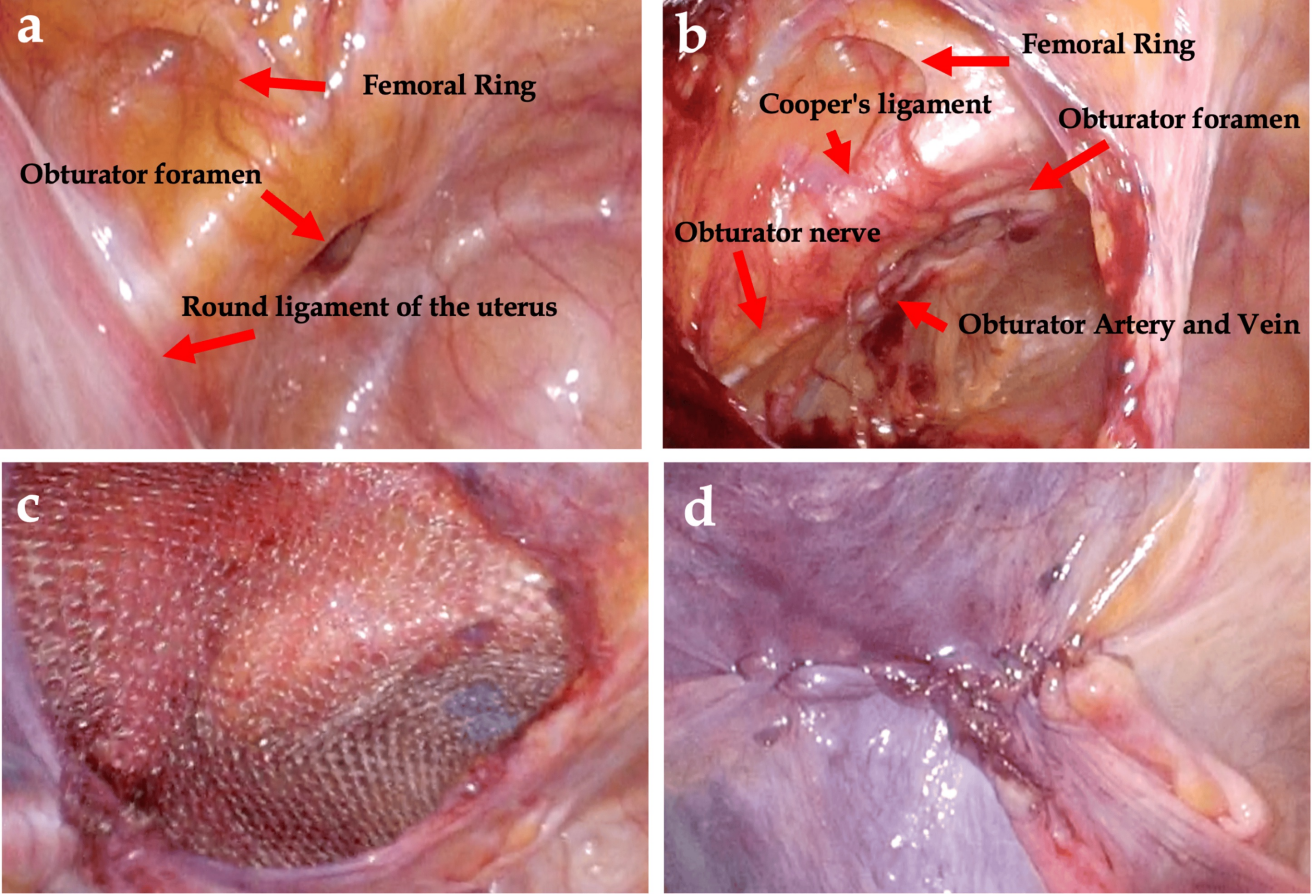
Intraoperative findings: (a) left obturator hernia and femoral hernia. (b) The peritoneum was dissected from the left obturator foramen and femoral hernia using Cooper's ligament as a landmark to create space for inserting the mesh. (c) Using the inversion technique, the 3D mesh was placed over the obturator and femoral hernias with a sufficient margin. (d) The peritoneum was sutured.

**Figure 4:**
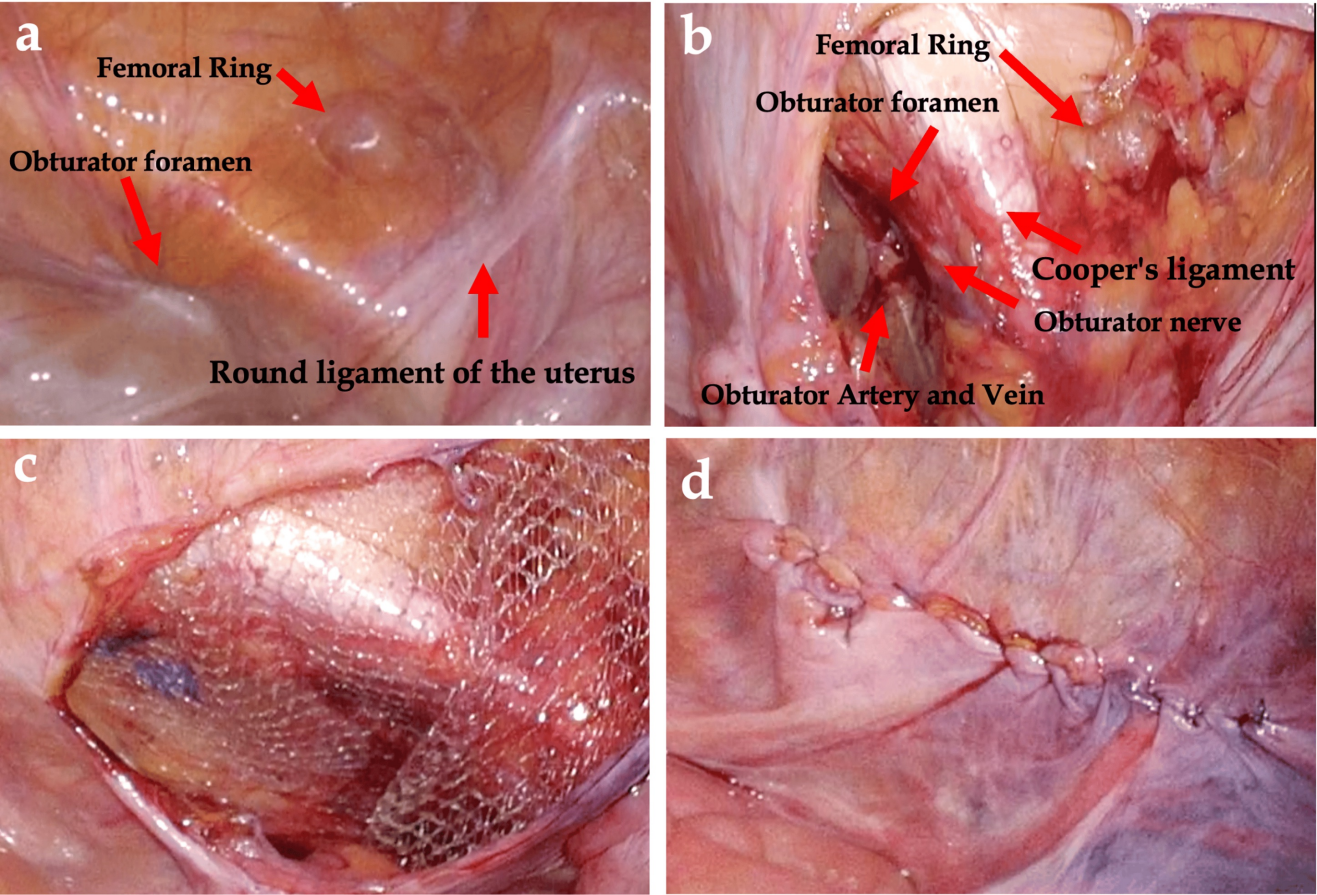
Intraoperative findings: (a) right obturator hernia and femoral hernia. (b) The peritoneum was dissected from the left obturator foramen and femoral hernia using Cooper's ligament as a landmark to create space for inserting the mesh. (c) Using the inversion technique, the 3D mesh was placed over the obturator and femoral hernias with a sufficient margin. (d) The peritoneum was sutured.

On day three after admission, the patient started to take food orally and was discharged. One month later, the patient was readmitted, and TAPP was performed on the OH. Intraoperative findings were bilateral OH and femoral hernia. The peritoneum was removed on both sides, and the 3D mesh was placed inside out and fixed with a stapler, after which the peritoneum was closed with a continuous suture (Figure 3: left side; Figure 4: right side).

The operative time was two hours and one minute, and blood loss was 1 g. The postoperative course was uneventful, and the patient was discharged on postoperative day three without complications.

## Discussion

OH mostly occurs in older women and women after pregnancy, due to the increased pelvic width, larger obturator canal, and weakness of pelvic tissues [1]. The term “little old ladies’ hernia” has been coined because of the predisposition to OH in older women caused by atrophy and loss of pre-peritoneal fat around obturator vessels in the canal [1]. OH commonly presents as an idiopathic intestinal obstruction in elderly underweight women with no history of abdominal surgery. The condition may be immediate or intermittent, but it is the initial complaint in almost 90% of cases [6]. There are also signs of strangulated hernia.

A Howship-Romberg sign reflects pain in the inner thigh, and this may be worsened by adduction, extension, and medial rotation of the thigh. This pain is due to compression of the cutaneous branch of the obturator nerve. However, this sign is found in <50% of cases of OH. A Hannington-Kiff sign occurs in a case with an intact patellar reflex with loss of the thigh adductor reflex, due to compression of the obturator nerve weakening the adductor muscles [7]. In our case, neither a Howship-Romberg sign nor a Hannington-Kiff sign was observed.

**Figure 5:**
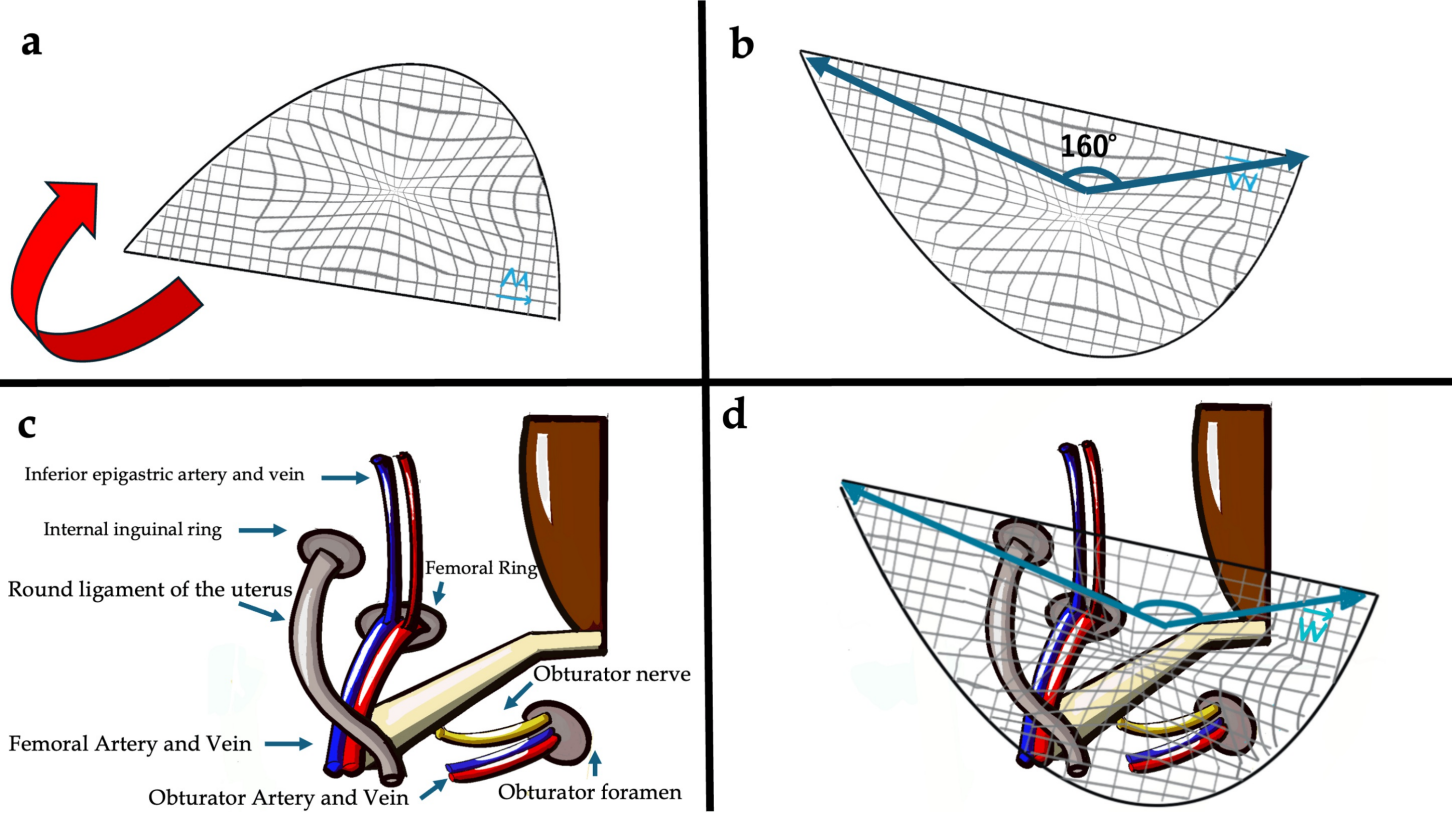
(a) Schema of 3D mesh layout. By inverting the 3D mesh in the direction indicated by the arrow, it transforms into (b). (b) Schema of use of 3D mesh using the inversion technique. (c) Schema of the anatomy of the groin area. (d) Schema showing 3D mesh placed using the inversion technique in the inguinal region. Image credit: the authors

Delayed diagnosis of OH may cause bowel ischemia to develop, necessitating bowel resection [8]. CT is useful for diagnosing OH, and diagnostic pre-operative CT can reduce postoperative complications [8]. Early diagnosis of OH may allow manual reduction, which in turn may permit elective surgery [8]. Surgical repair is the gold standard for the treatment of OH, and there are several surgical approaches [3], but the closeness of the obturator nerve to the obturator defect limits the view in the laparotomy operative field [4]. Since most patients with OH are elderly and have many comorbidities, the laparoscopic approach is a safe and minimally invasive method that can be performed when emergency surgery is not required [9]. Also compared to non-mesh repairs, mesh repairs are associated with less hernia recurrence, faster return to daily life, and shorter surgery times [10]. TAPP for OH repair allows better observation of the obturator canal and reduces postoperative complications [4], while laparoscopic surgery can detect potential hernia [11]. Sasa et al. proposed a 3D mesh inversion technique in which the mesh is placed with the anterior and posterior sides reversed [5]. In pelvic 3D-CT, the angle between the obturator fascial plane and the Hesselbach triangle at Cooper's ligament in elderly thin women has been found to be about 160°. Measurement of the curvature along the long axis of the 3D mesh revealed that the angle was nearly the same (Figures 5a, 5b). By inverting the 3D mesh, it was possible to cover the OH orifice in an anatomically natural manner (Figures 5c, 5d).

Moreover, because the shape of the mesh could be maintained, it was possible to coat the hernia orifice by fixing the mesh at a minimum level [5]. On the other hand, due to its shape, it is difficult to cover the inverted mesh in cases of combined external inguinal hernia.

In this case, we were able to detect multiple potential hernias by performing elective laparoscopic surgery to release the incarcerated OH. Because the patient had both an obturator and femoral hernias, we were able to make the mesh anatomically fit using the 3D mesh inversion technique. This is the first report of the use of elective TAPP using a 3D mesh inversion technique for bilateral OH and femoral hernia after incarcerated OH reduction.

## Conclusions

The surgical technique for incarcerated OH is controversial. However, if the incarceration can be manually relieved, laparoscopic surgery can be used to observe the details of multiple hernias, and the use of a 3D mesh inversion technique allows for anatomical mesh fitting. On the other hand, this method is difficult to use in cases where an OH and an external inguinal hernia are combined. However, it can be used in cases where a femoral hernia and an internal inguinal hernia are combined, so careful consideration should be given before using this method.
